# Circular RNA expression profile of lung squamous cell carcinoma: identification of potential biomarkers and therapeutic targets

**DOI:** 10.1042/BSR20194512

**Published:** 2020-04-28

**Authors:** Yawei Wang, Haiyan Zhang, Jian Wang, Bei Li, Xiuwen Wang

**Affiliations:** 1 Department of Medical Oncology, Qilu Hospital, Shandong University, Jinan, Shandong 250012, China; 2 Department of Otorhinolaryngology Head and Neck Surgery, Shandong Provincial ENT Hospital, Shandong University, Jinan, Shandong 250021, China

**Keywords:** biomarkers, circRNAs profile, hsa_circ_0014235, hsa_circ_0025580, LUSC

## Abstract

Emerging evidences indicated that exosomal circular RNAs (circRNAs) could serve as diagnostic biomarkers for cancers. However, the expression profiles and clinical significance of circRNAs in lung squamous cell carcinoma (LUSC) remain largely unknown. Herein, we analyzed circRNAs expression profile in six pairs of plasma exosome samples of LUSC patients using high-throughput sequencing. A total of 252 differentially expressed exosomal circRNAs were identified, including 133 up-regulated circRNAs and 119 down-regulated circRNAs. Subsequently, the circRNAs–miRNAs–mRNAs interaction network was built to investigate potential function of circRNAs. Three up-regulated circRNAs (hsa_circ_0014235, hsa_circ_0025580 and hsa_circ_0026403) were implied to participate in cancer-related pathways. QRT-PCR experiment confirmed the up-regulation of hsa_circ_0014235 and hsa_circ_0025580. Finally, clinical studies indicated that hsa_circ_0014235 and hsa_circ_0025580 could serve as novel diagnostic biomarkers for LUSC. Taken together, our study revealed exosomal circRNAs expression profile in LUSC for the first time and showed the important diagnostic potential for circRNAs in LUSC.

## Introduction

Lung cancer is one of the most widely spread cancers world-wide, accounts for the highest rate of cancer-related mortality [1]. Lung squamous cell carcinoma (LUSC) is one of the major subtypes of non–small cell lung cancer (NSCLC), accounting for 20% of NSCLC cases [2,3]. The 5-year survival rate for LUSC patients is poor and the targeted therapies available for LUSC are limited [4,5]. Therefore, it is urgently needed to explore the potential molecular biomarkers for LUSC diagnosis.

Exosomes are small membranous vesicles, serve as important carriers among cells and transmit proteins, RNAs and lipids from cell into the extracellular space [6,7]. Recent evidence demonstrated that exosome-derived RNAs may involve in cell proliferation, cancer progression and drug resistance [8]. Circular RNAs (circRNAs) are emerging as a novel class of non-coding RNAs and back-spliced from downstream 5′ site to an upstream 3′ site with a special loop [9]. The structure of circRNA is more stable than linear RNAs and is resistant to RNase R degradation or RNA exonuclease digestion; this makes it a promising clinical biomarker for disease [10]. For example, Liu et al. found that exosomal circRNA_100284 was involved in malignant transformation of human hepatic cells [11]. Several recent studies indicated that circRNAs performs its function in cancers by acting as sponges of microRNAs (miRNAs), including glioma, breast cancer and lung cancer [12,13]. However, the role of exosomal circRNAs in LUSC patients has not been reported.

In the present study, we performed high-throughput sequencing and bioinformatics program to analyze the alteration of circRNAs expression in plasma exosome samples from LUSC patients for the first time. The circRNAs–miRNAs–mRNAs interaction network was then constructed for top 10 differentially expressed circRNAs. Three up-regulated circRNAs that involved in cancer-related pathways were validated by qRT-PCR experiment. Further medical studies showed that certain circRNAs may serve as novel diagnostic biomarkers for LUSC.

## Materials and methods

### Patient samples and exosome isolation

A total of 30 pairs of LUSC patient samples and normal control samples were collected from Qilu Hospital with participant’s consent. None of the participants had received chemotherapy treatment before. The study was approved by Ethics Committee of Qilu Hospital and was in accordance with the Helsinki Declaration. For exosome isolation, the process was performed according to the instruction of Hieff exosome isolation kit (Shanghai, China). The plasma was separated in a centrifuge tube and centrifuge at 3000 rcf for 10 min (4°C). The clear supernatant was then transferred to another labeled tube and the pelleted exosomes were re-suspended in 1× PBS, stored at −80°C. The Bradford assay was used to measure the concentration of exosomes.

### Illumina sequencing of circRNAs

Total RNA was generated from six pairs of exosome samples (LUSC and control) using Trizol (Invitrogen, CA, U.S.A.) following the instructions. The integrity and concentration of RNAs were then examined and the results were met the standards for subsequent experiments: (RIN) ≥ 7.0; 28S:18S ratio ≥ 1.5. After removal of rRNA, the total RNA was digested with RNase R to remove linear RNAs. The cDNA library was prepared according to Illumina TruSeq library preparation instruction. CircRNA sequencing was conducted on an Illumina HiSeq sequencer (Illumina, CA, U.S.A.).

### Bioinformatics analyses of sequencing data

The raw sequencing reads were evaluated by FastQC software. After filtering out low-quality reads, the remaining reads were aligned to GRCH38 genome using TopHat2 software [14]. Reads that were left after alignment were subjected to CIRCexplorer, MapSplice and CircRNA_finder software to identify circRNAs [15]. The differentially expressed circRNAs were analyzed by R package Limma with fold change ≥ 2 and corrected *P*-value < 0.01. The pathway enrichment analysis was performed by KOBAS software [16].

### Construction of circRNAs–miRNAs–mRNAs network

We predicted the target miRNAs of top 10 differentially expressed circRNAs using miRanda and TargetScan algorithms [17]. The miRNAs obtained from two algorithms were intersected. The target genes of miRNAs were predicted by miRWalk 3.0 database [18], which is a comprehensive atlas of predicted and validated miRNAs–targets interactions. The circRNAs–miRNAs–mRNAs network was constructed by Cytoscape software.

### QRT-PCR quantification of circRNAs

To validate the reliability of sequencing data and explore the circRNAs expression trend in LUSC, qRT-PCR experiment was conducted. Firstly, total RNAs were digested by RNase R to remove linear RNAs. Primers for circRNAs were designed by crossing back-spliced junction sites and were synthesized by Biotech company (Biotech, Beijing, China). The real-time PCR analysis was conducted using SYBR kit (Takara, Japan) on Bio-Rad CFX96 detection system, and glyceraldehyde 3-phosphate dehydrogenase (GAPDH) was used as internal control.

### Statistical analyses

All statistical analyses in the present study were performed using R software. The categorical data were analyzed by Chi-square test and setting the significance threshold as *P*-value < 0.05. Data were presented as the means ± standard error of the mean and determinations were performed at least three times. *P*-value < 0.05 was considered as statistically significant.

## Results

### CircRNA profiling in plasma exosome from LUSC

**Figure 1:**
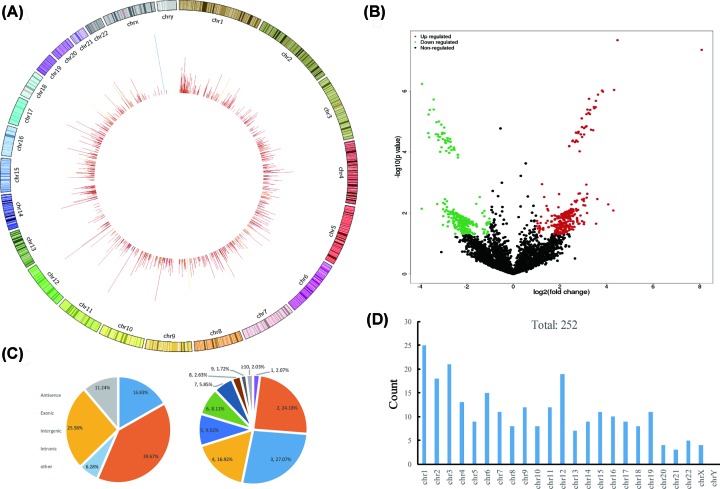
CircRNA profiling in LUSC (**A**) Genome distribution of identified circRNAs in LUSC exosome samples. (**B**) Differentially expressed exosomal circRNAs in LUSC compared with control. Red: up-regulated circRNAs. Green: down-regulated circRNAs. (**C**) Category and exon number statistics of differentially expressed exosomal circRNAs. (**D**) Genome distribution of differentially expressed exosomal circRNAs.

After RNase R digestion of linear RNAs, six pairs of plasma exosome samples from LUSC patients and normal controls were subjected to Illumina sequencing. We analyzed the exosomal circRNAs using CIRCexplorer, MapSplice and CircRNA_finder software. A total of 2593 circRNAs were identified in plasma samples (data not shown). The distribution pattern of these exosomal circRNAs were investigated. As shown in Figure 1A, most of the circRNAs were back-spliced from chr1, chr7 and chr14. By comparing circRNAs expression between LUSC and control, 252 differentially expressed circRNAs were identified with fold change ≥ 2 and corrected *P*-value < 0.01 (Figure 1B), among which 133 circRNAs were up-regulated and 119 circRNAs were down-regulated. These differentially expressed circRNAs were mostly derived from exonic region, containing a variable number of exons (Figure 1C). Besides, genome distribution analysis showed these differentially expressed circRNAs were mainly distributed in chr1, chr3 and chr12 (Figure 1D).

### Top expressed exosomal circRNAs and circRNAs–miRNAs–mRNAs interaction network

**Figure 2:**
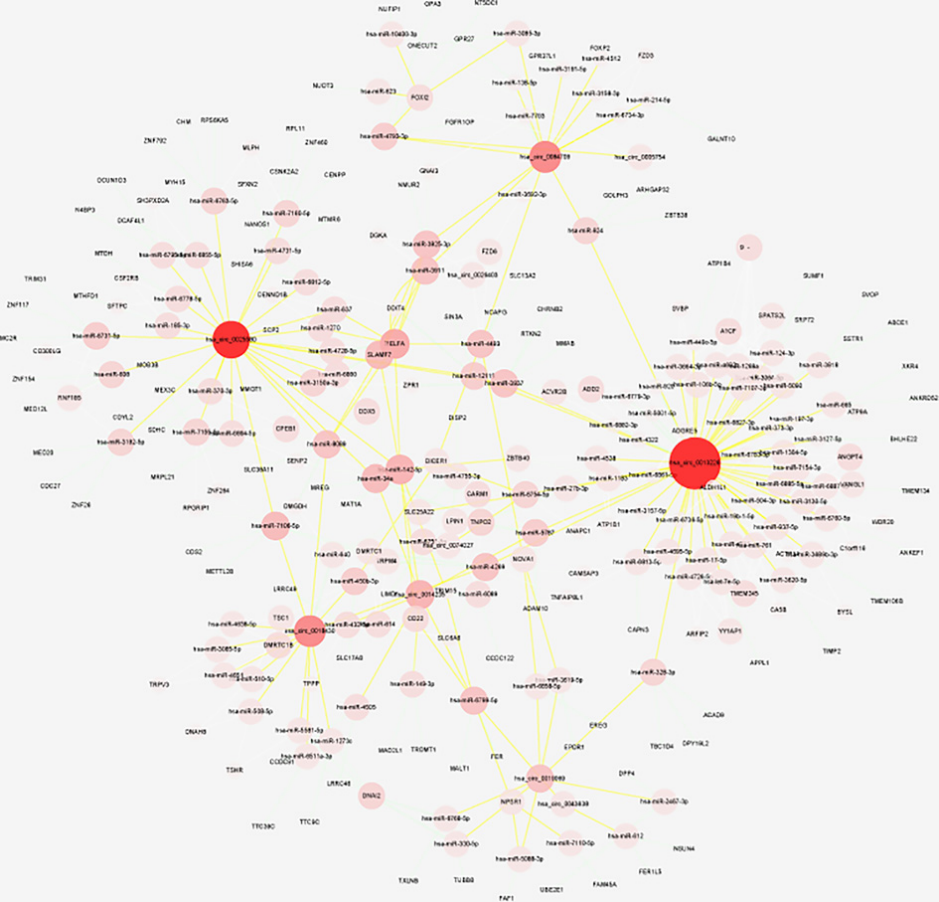
The circRNAs–miRNAs–mRNAs interaction network of top 10 dysregulated circRNAs

By screening expression level and fold change of exosomal circRNAs, we listed top 10 differentially expressed circRNAs and their detailed information in Table 1. These differentially expressed circRNAs may play important roles in the progression of LUSC. Moreover, circRNAs–miRNAs–mRNAs interaction network was built based on top 10 differentially expressed circRNAs using miRanda, TargetScan algorithms and miRWalk 3.0 database (Figure 2). KEGG pathway analyses of targeted mRNAs implied that three circRNAs (hsa_circ_0014235, hsa_circ_0025580 and hsa_circ_0026403) might involve in regulation of cell cycle, Jak−STAT signaling pathway and Wnt signaling pathway (Supplementary Figure S1).

**Table 1:** Top 10 differentially expressed exsomal circRNAs in LUSC samples compared with control

CircRNA	Fold change (abs)	*P*-value	Regulation	Chromosome	Host gene
hsa_circ_0018430	8.56	0.0005	up	chr10:60562836-60563002	BICC1
hsa_circ_0014235	8.03	0.0079	up	chr1:153536209-153536363	S100A2
hsa_circ_0013226	6.81	0.0037	down	chr1:94668473-94674460	ARHGAP29
hsa_circ_0025580	6.86	0.0008	up	chr12:21028168-21229503	SLCO1B3
hsa_circ_0005754	6.49	0.0083	down	chr15:33076591-33076875	FMN1
hsa_circ_0043638	6.11	0.0004	up	chr17:39775691-39779284	KRT17
hsa_circ_0019069	5.98	0.0069	down	chr10:91195816-9122238	SLC16A12
hsa_circ_0084709	5.76	0.0002	down	chr8:68931783-68984805	PREX2
hsa_circ_0074027	5.38	0.0090	up	chr5:134363423-134369964	PITX1
hsa_circ_0026403	4.36	0.0092	up	chr12:52843252-52843417	KRT6B

### QRT-PCR validation of key circRNAs

**Figure 3:**
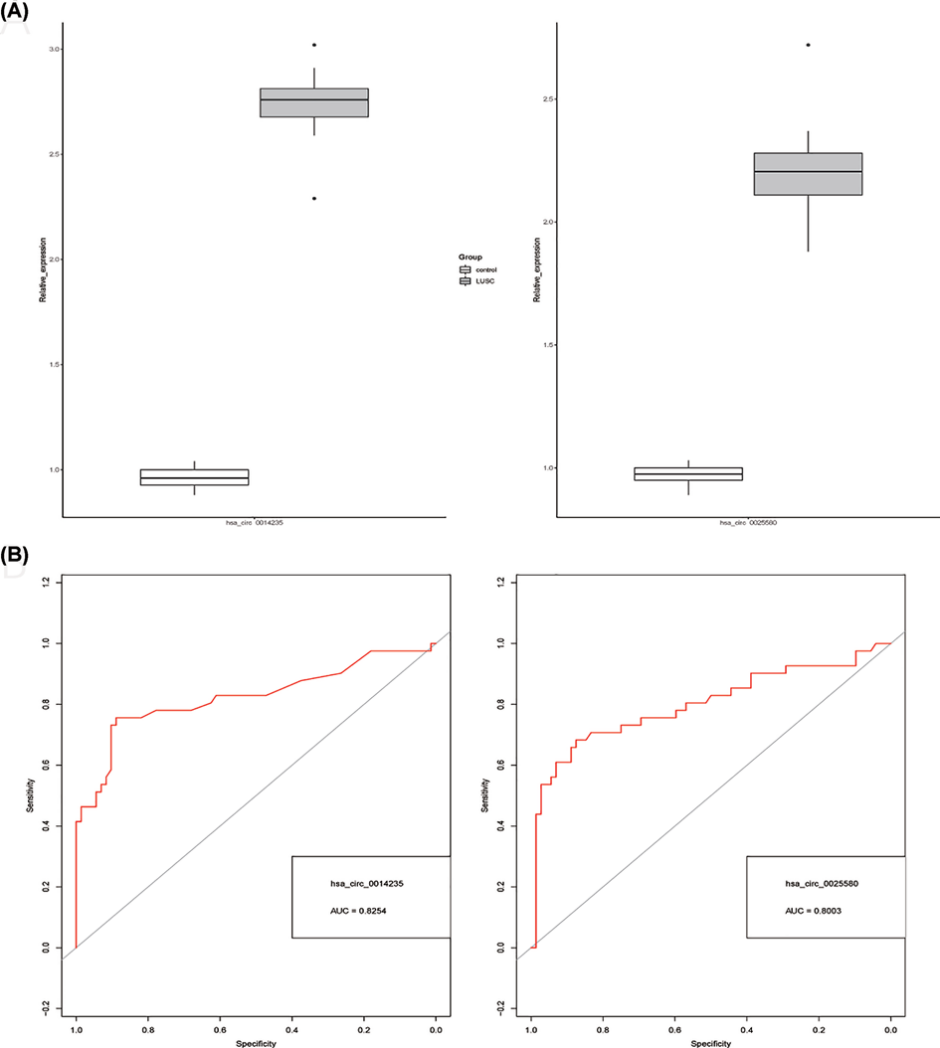
Hsa_circ_0014235 and hsa_circ_0025580 served as potential biomarkers for LUSC diagnosis (**A**) Up-regulation of hsa_circ_0014235 and hsa_circ_0025580 in LUSC exosome was validated by qRT-PCR (sample size: 30; *P* < 0.05). (**B**) ROC curve analyses of hsa_circ_0014235 and hsa_circ_0025580.

To further detect the abundance of hsa_circ_0014235, hsa_circ_0025580 and hsa_circ_0026403 in plasma, we performed qRT-PCR validation in 30 pairs of plasma exosome samples of LUSC patients and normal controls. As shown in Figure 3A, the up-regulation of hsa_circ_0014235 and hsa_circ_0025580 was detected in LUSC patients compared with controls (fold change: 2.78 and 2.25, respectively; *P* < 0.05), consistent with previous sequencing data (fold change: 6.86 and 8.03, respectively). However, hsa_circ_0026403 expression did not show a significant increase in LUSC group (fold change: 1.17; *P* = 0.643; data not shown).

### Hsa_circ_0014235 and hsa_circ_0025580 served as potential biomarkers for LUSC diagnosis

To measure the clinical significance of hsa_circ_0014235 and hsa_circ_0025580 in LUSC patients, we analyzed the Pearson correlation between circRNAs expression and LUSC clinical outcomes. As shown in Table 2, increased expression of hsa_circ_0014235 and hsa_circ_0025580 was strongly correlated with higher TNM stage (*P*-value: 0.028 and 0.019, respectively) and larger tumor size (*P*-value: 0.026 and 0.022, respectively). To further assess the diagnostic potential of two circRNAs in LUSC, ROC curve analyses were conducted. The results showed that there were acceptable diagnostic values for hsa_circ_0014235 (AUC = 0.8254, 95% CI: 0.762–0.889) and hsa_circ_0025580 (AUC = 8003, 95% CI: 0.741–0.862) in LUSC samples (Figure 3B), suggesting the high diagnostic value for LUSC.

**Table 2:** Expression of circRNAs and clinical features of LUSC patients (*n* = 30)

Clinical index	hsa_circ_0014235 expression		*P*-value	hsa_circ_0025580 expression		*P*-value
	High	Low		High	Low	
**Age**			0.835			0.798
<60	6	8		7	7	
≥60	9	7		9	7	
**Gender**			0.501			0.412
Male	7	9		6	8	
Female	8	6		10	6	
**Smoking**			0.378			0.266
Yes	9	7		8	6	
No	6	8		8	8	
**TNM stage**			0.008			0.009
I-II	4	7		4	8	
III-IV	11	8		12	6	
**Tumor size (cm)**			0.006			0.002
≤3	3	7		2	7	
>3	12	8		14	7	

## Discussion

Recent studies indicated that exosomes have significant influences on pathophysiologic processes of diseases, by acting as important messengers in cell communication and carrying some of proteins, RNAs and other components [6]. With the development of high-throughput sequencing technology and bioinformatics algorithm, circular RNAs have been identified in human tissues, with cell-type or tissue-specific expression pattern and high stability [9]. In recent years, exosomal circRNAs serve as potential biomarker in disease diagnosis and have drawn great attention [8]. For example, a previous study found that the serum exosomal hsa-circ-0004771 expression was notably increased in colorectal cancer patients and could serve as novel diagnostics biomarker [19]. An exosomal circRNA, circPTGR1 was found to promote metastasis of hepatocellular carcinoma [20]. In the present study, we investigate the exosomal circRNAs expression pattern in LUSC patient using high-throughput sequencing. A total of 252 circRNAs were identified to be differentially expressed between LUSC tissue samples and control samples. These circRNAs were mostly derived from exonic region and mainly distributed in chr1, chr3 and chr12.

Moreover, we screened out top 10 differentially expressed circRNAs with high expression level and large fold change. Further exploration of these top expressed exosomal circRNAs may help us better understand their role in LUSC progression. Besides, increasing evidences indicated that circRNAs could function as sponges of microRNAs and form a large class of post-transcriptional regulator [21,22]. The constructed circRNAs–miRNAs–mRNAs interaction network provide a new sight into potential function and mechanism of circRNAs in LUSC. For these mRNAs that indirectly targeted by circRNAs, we performed KEGG pathway enrichment analyses and found that three circRNAs (hsa_circ_0014235, hsa_circ_0025580 and hsa_circ_0026403) may involve in cancer-related pathway. Further study is needed to investigate the underlying mechanism of these circRNAs in LUSC development.

The abundance of three circRNAs was validated by qRT-PCR and the results showed that expression of hsa_circ_0014235 and hsa_circ_0025580 were notably increased in LUSC exosome samples (*n* = 30); while no significant change was observed in hsa_circ_0026403 expression, it was likely to be driven by sequencing inaccuracy. The Chi-square test indicated the positive correlation between circRNAs expression and TNM stage. Further ROC analyses indicated that there were acceptable diagnostic values for hsa_circ_0014235 and hsa_circ_0025580.

In conclusion, our study explored the exosomal circRNAs expression pattern in LUSC patients for the first time. Moreover, hsa_circ_0014235 and hsa_circ_0025580 could serve as novel diagnostic biomarkers for LUSC.

## Supplementary Material

Supplementary Figure S1

## Abbreviations

circRNA: circular RNA
GAPDH: glyceraldehyde 3-phosphate dehydrogenase
LUSC: lung squamous cell carcinoma
miRNA: microRNA
NSCLC: non–small cell lung cancer
