# Supplementary material for: Circular RNA expression profile of lung squamous cell carcinoma: identification of potential biomarkers and therapeutic targets
Source: Biosci Rep. 2020 Apr 28;40(4):BSR20194512. doi: 10.1042/BSR20194512 (PMC7189478; doi:10.1042/BSR20194512)
Supplement: Supplementary Figure S1 [file BSR-2019-4512_supp.pdf]

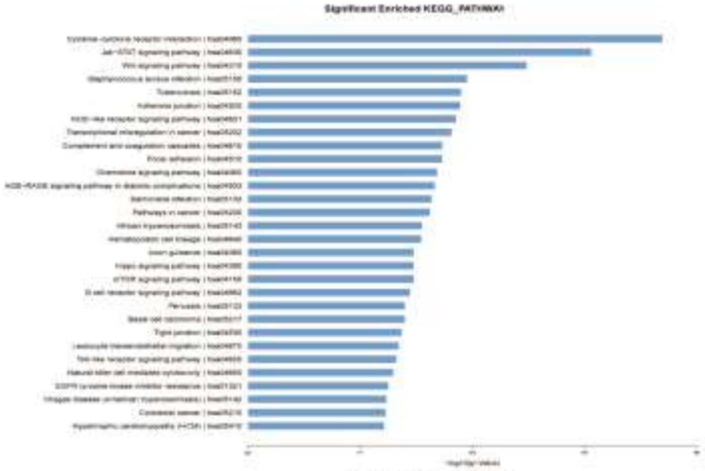
Significant Enriched KEGG_PATHWAY

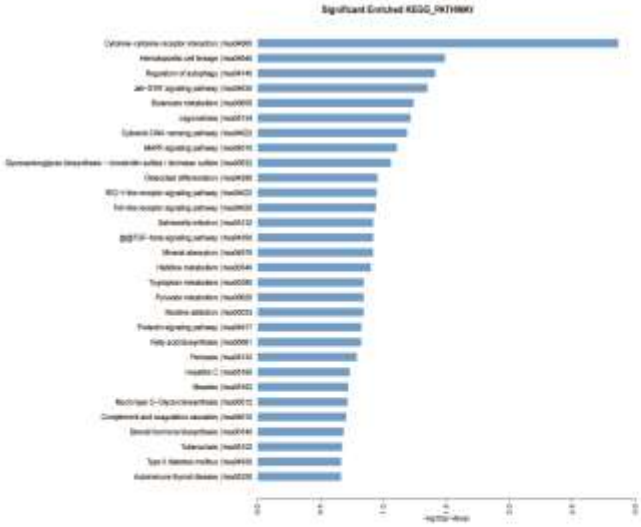
Significant Enriched KEGG_PATHWAY

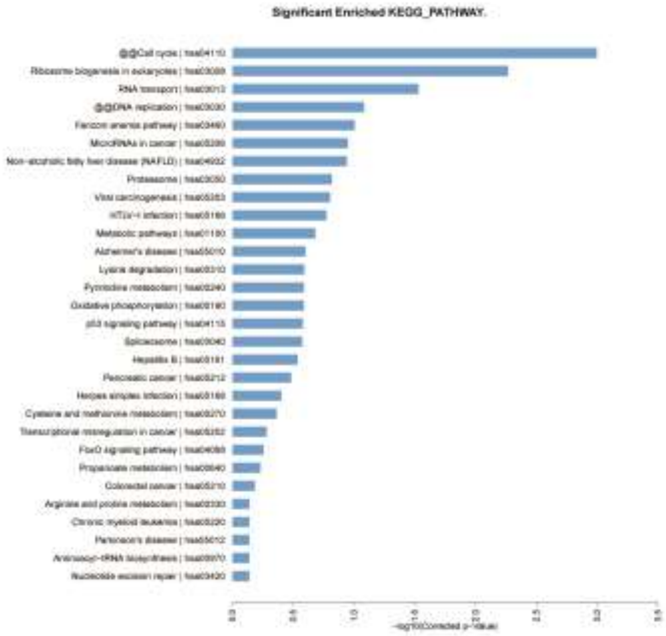
Significant Enriched KEGG_PATHWAY.
@@Cell cycle | hsa04110
Ribosome biogenesis in eukaryotes | hsa03008
RNA transport | hsa03013
@@DNA replication | hsa03030
Fanconi anemia pathway | hsa03460
MicroRNAs in cancer | hsa05206
Non-alcoholic fatty liver disease (NAFLD) | hsa04932
Proteasome | hsa03050
Viral carcinogenesis | hsa05203
HTLV-I infection | hsa05166
Metabolic pathways | hsa01100
Alzheimer's disease | hsa05010
Lysine degradation | hsa00310
Pyrimidine metabolism | hsa00240
Oxidative phosphorylation | hsa00190
p53 signaling pathway | hsa04115
Spliceosome | hsa03040
Hepatitis B | hsa05161
Pancreatic cancer | hsa05212
Herpes simplex infection | hsa05168
Cysteine and methionine metabolism | hsa00270
Transcriptional misregulation in cancer | hsa05202
FoxO signaling pathway | hsa04068
Propanoate metabolism | hsa00640
Colorectal cancer | hsa05210
Arginine and proline metabolism | hsa00330
Chronic myeloid leukemia | hsa05220
Parkinson's disease | hsa05012
Aminoacyl-tRNA biosynthesis | hsa00970
Nucleotide excision repair | hsa03420
-log10(Corrected p-Value)

Supplementary Figure 1. KEGG analyses of indirectly targeted mRNAs of hsa_circ_0014235, hsa_circ_0025580 and hsa_circ_0026403 in circRNAs-miRNAs-mRNAs network.
